# Mixed Gels of Fresh White Cheese (Twaróg) with Gelatin

**DOI:** 10.3390/molecules31213860

**Published:** 2026-10-26

**Authors:** Marta Tomczyńska-Mleko, Bartosz G. Sołowiej, Konrad Terpiłowski, Katsuyoshi Nishinari, Stanisław Mleko

**Affiliations:** 1 Institute of Plant Genetics, Breeding and Biotechnology, Faculty of Agrobioengineering, University of Life Sciences in Lublin, 20-950 Lublin, Poland; 2 Department of Dietetics, Faculty of Food Sciences and Biotechnology, University of Life Sciences in Lublin, 20-704 Lublin, Poland; 3 Department of Dairy Technology and Functional Foods, Faculty of Food Sciences and Biotechnology, University of Life Sciences in Lublin, 20-704 Lublin, Polandstanislaw.mleko@up.edu.pl (S.M.); 4 Department of Interfacial Phenomena, Maria Curie Skłodowska University, 20-031 Lublin, Poland; 5 Glyn O. Phillips Hydrocolloid Research Centre, School of Bioengineering and Food Science, Hubei University of Technology, Wuhan 430068, China; katsuyoshi.nishinari@gmail.com

**Keywords:** casein, gelatin, rheology, IR-ATR, surface roughness, microstructure

## Abstract

Mixed gels based on fresh white cheese (twaróg) and type A gelatin were prepared to investigate their rheological, microstructural, surface, and storage-related properties and to examine possible interactions between casein and gelatin. Selected gels underwent a blending/aeration treatment that simultaneously reduced particle/cluster size and incorporated air. Increasing gelatin concentration was associated with stronger viscoelastic and textural responses. Because total solids were held constant, increasing the gelatin concentration also decreased the casein fraction; therefore, the observed changes reflect changes in the relative proportions of the two protein components as well as the addition of gelatin. ATR-FTIR spectra showed band shifts consistent with changes in the local molecular environment, but these shifts do not establish specific casein–gelatin interactions or changes in casein secondary structure. The blending/aeration treatment was associated with changes in gel microstructure, lower surface roughness, and a lower water contact angle. Turbiscan profiles showed optical changes during 14 days of refrigerated storage. The milled gel containing 2% gelatin had the lowest final TSI among the measured profiles; because only one independently prepared sample per formulation was measured, these comparisons are descriptive and do not establish statistically significant differences in stability or product shelf life. The mixed gels may have potential for refrigerated curd-cheese snack products, although further product-scale validation is required.

## 1. Introduction

Fresh white cheese (twaróg; pronounced “tfa-rook”) is a traditional acid-coagulated dairy product that contains predominantly casein proteins. The protein content of low-fat twaróg can reach 20% (*w*/*w*). Twaróg is produced by natural acidification with lactic acid bacteria without the use of an enzyme, resulting in a characteristic granular texture and sour taste. It is traditionally used in Eastern Europe to prepare cheesecake, which differs from many other types of cheesecake in sweetness and in the acid or chymosin coagulation process used. Recently, curd cheese has also been used to produce high-protein products. For example, fresh curd cheese has been ground with whey protein isolate and heated [1]. Curd cheese is also used to produce curd-bar snacks, which are usually coated with dark or milk chocolate and may contain a jam or fruit filling. The main component of these snacks is curd cheese mixed with sugar, sweeteners, or other ingredients, milled into a homogeneous mass, and pressed into bars. The bars are usually glazed with chocolate, cooled, and packaged. Because these products contain active lactic acid bacteria, their shelf life without refrigeration is short, and their texture may become too soft at room temperature. The aim of this study was to evaluate the effects of gelatin addition on the hardness and other properties of mixed twaróg–gelatin gels as a basis for a potential curd-bar application. The study explores a possible application rather than demonstrating a finished product; industrial implementation would require development and validation of a dedicated production process.

Surprisingly, a search of the scientific literature shows a lack of publications on solid casein/gelatin gels. Pang et al. [2] published an excellent, frequently cited paper on the effect of gelatin addition on the rheological and microstructural properties of acid milk protein gels. Gelatin was added to milk before the coagulation process. In the acidification stage performed at a temperature at which gelatin remained liquid (45 °C), its presence resulted in a lower storage modulus of the curd and a more heterogeneous microstructure with larger milk protein clusters. As a matter of fact, cooling of the curd with gelatin led to an increase in elasticity. Higher gelatin concentrations resulted in the formation of strand-like structures. In the heating stage (again 45 °C), gelatin strands melted and the storage modulus of the mixed gels reverted to the final value of the acidification stage. Additionally, gelatin enhanced the water-holding capacity of the gels, without a significant increase in gel firmness. The authors used type B gelatin obtained from beef skin with an isoelectric point of ca. 5.0 [2]. This may explain the absence of electrostatic interactions between casein and gelatin in the acidification process at pH 4.6.

Gelatin is obtained from beef bones and hides, pork skin and bones, as well as calf skin. The process involves the chemical hydrolysis of collagen, followed by heating to denature the collagen molecules into gelatin. There are two types of gelatin: A and B. Type A gelatin is obtained by the acid pretreatment of physiologically young forms of collagen (usually from pig skin), which contain a high proportion of acid- and heat-labile cross-links [3]. Type B gelatin is produced by the more severe alkaline pretreatment of the more highly cross-linked collagen from beef bone and hides [4]. The isoelectric points of both gelatin types are quite different. For type A gelatin, the isoelectric point is in the pH range 6–9, and for type B the isoelectric point is close to that of casein: 4.8–5.2. In our research, gels were produced from casein curd cheese mixed with a commercially available food-grade gelatin commonly used in Poland (Dr. Oetker, Gdańsk, Poland). This was type A gelatin obtained by acid pretreatment of collagen from pig skin (150 kDa, food grade) [5]. The isoelectric point of the gelatin solution was determined using a Zetasizer [6]. It was 9.2, which is close to the value of 9.4 reported by Padilla et al. [6] for commercial type A porcine gelatin.

The working hypothesis was that type A gelatin could interact with casein under the conditions used to prepare the mixed gels and thereby affect their properties. The pH of twaróg is close to the isoelectric point of casein (approximately 4.6), whereas the gelatin used here had an isoelectric point of 9.2 and would therefore be expected to carry a net positive charge at the pH of the gels. These conditions make electrostatic interactions plausible, but they do not establish that such interactions occur in the mixed gels. Proteins have heterogeneous distributions of charged groups, and local interactions may occur even when the net charge is close to zero. Accordingly, the aim was to characterize the rheological, textural, microstructural, and surface properties of mixed casein–gelatin gels and to discuss possible molecular mechanisms cautiously.

## 2. Results and Discussion

### 2.1. Dynamic Oscillatory Measurements

**Figure 1:**
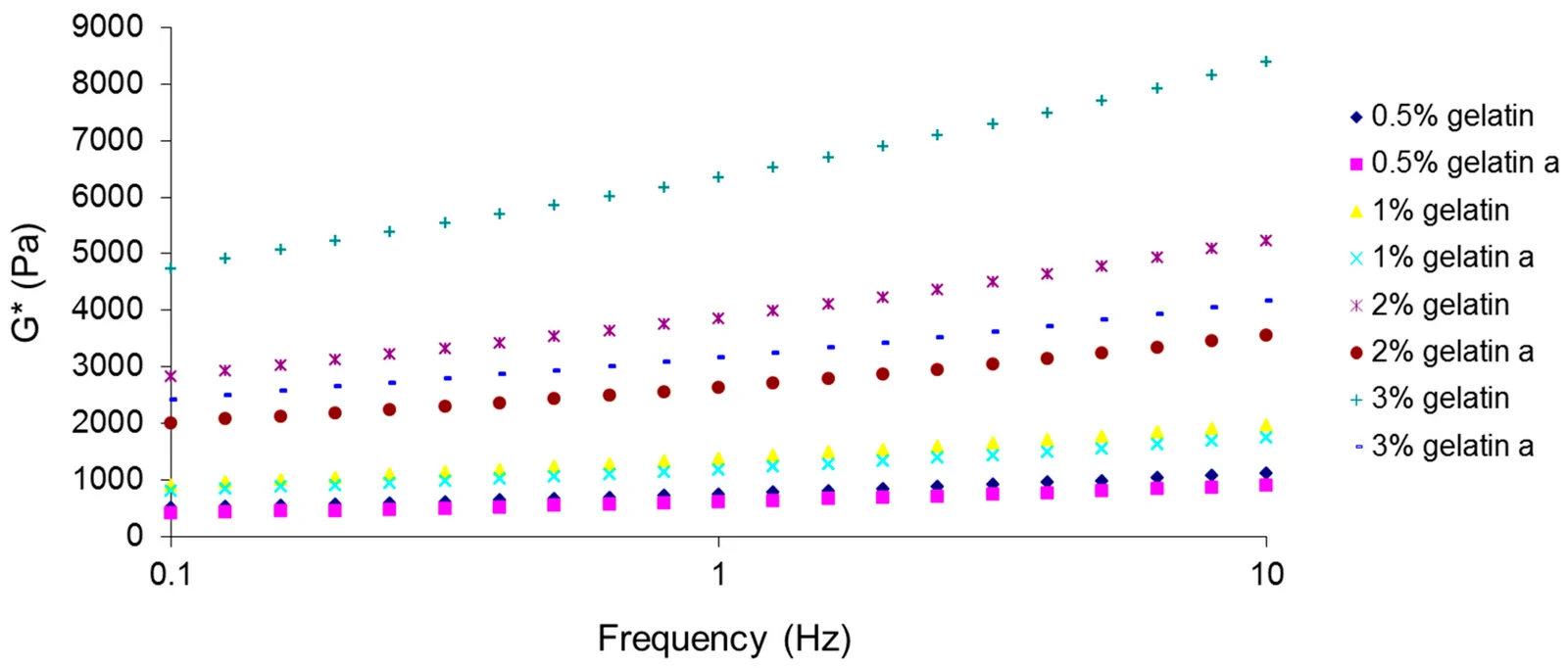
Influence of gelatin concentration on the frequency dependence of the complex modulus in mixed casein/gelatin gels (a—blended/aerated).

Figure 1 shows representative dynamic rheological responses of casein–gelatin mixed gels at different gelatin concentrations. Changes in the complex modulus (G*) were observed at different oscillation frequencies.

The complex modulus reflects the combined elastic and viscous response of a material. It increased with gelatin concentration, indicating stronger viscoelastic responses at higher gelatin levels. Blended/aerated samples showed lower complex modulus values than the corresponding milled samples. Because the treatment combined mechanical blending and air incorporation, the individual contributions of particle/cluster-size reduction and aeration cannot be separated in this study.

**Figure 2:**
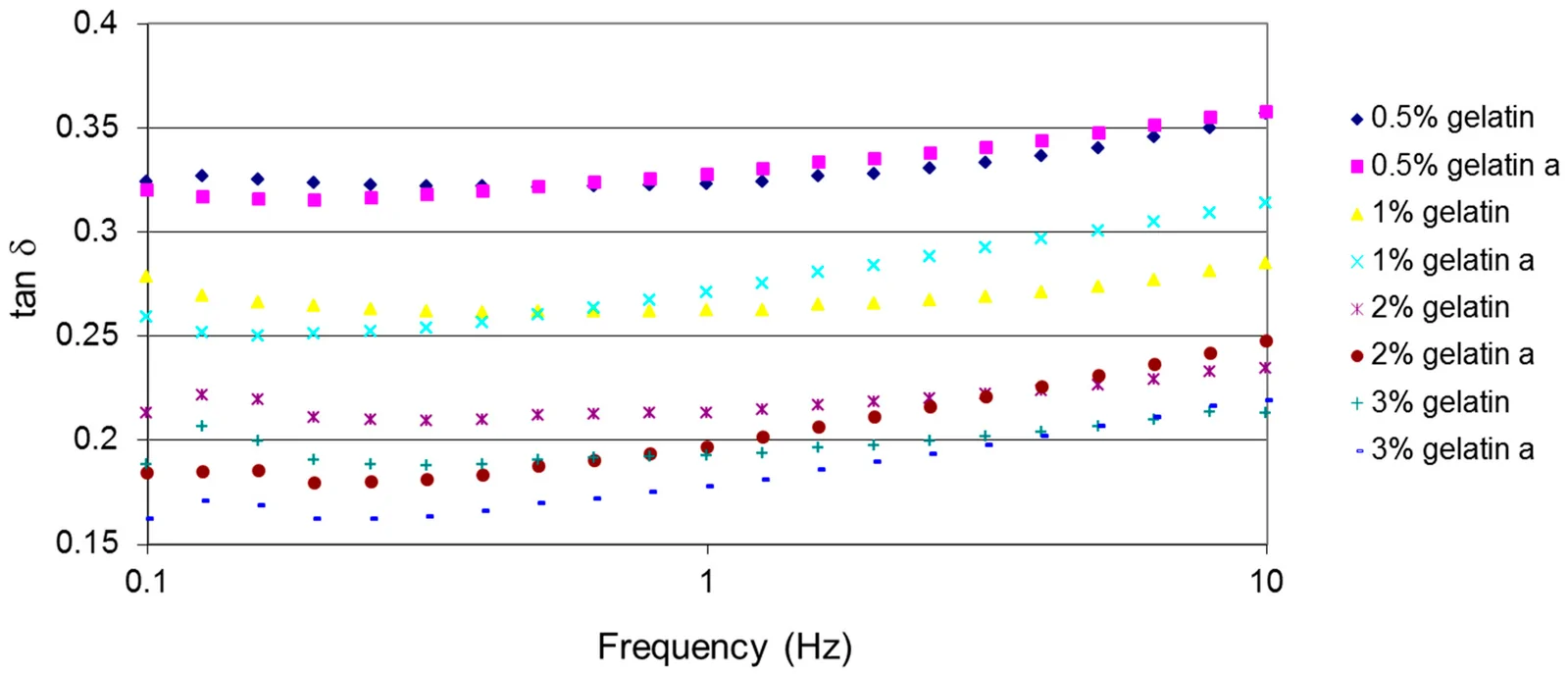
Influence of gelatin concentration on the frequency dependence of the loss tangent in mixed casein/gelatin gels (a—blended/aerated).

Figure 2 shows representative changes in the tan δ of mixed casein–gelatin gels across the tested frequencies. At 0.1 Hz, the blended/aerated gels had lower tan δ values, indicating a more elastic response at this frequency. As frequency increased to 10 Hz, the tan δ increased more in the blended/aerated gels, indicating a greater relative viscous contribution at higher frequencies. Air bubbles and changes in the structure caused by blending may contribute to this frequency-dependent response, but their individual effects were not isolated in the present experimental design. Wu et al. [7] reported that homogenized rice protein–starch mixtures had lower elasticity than untreated mixtures, illustrating that the effects of mechanical treatment depend on the composition and structure of the material.

### 2.2. Ultrasound Viscosity Measurements

**Figure 3:**
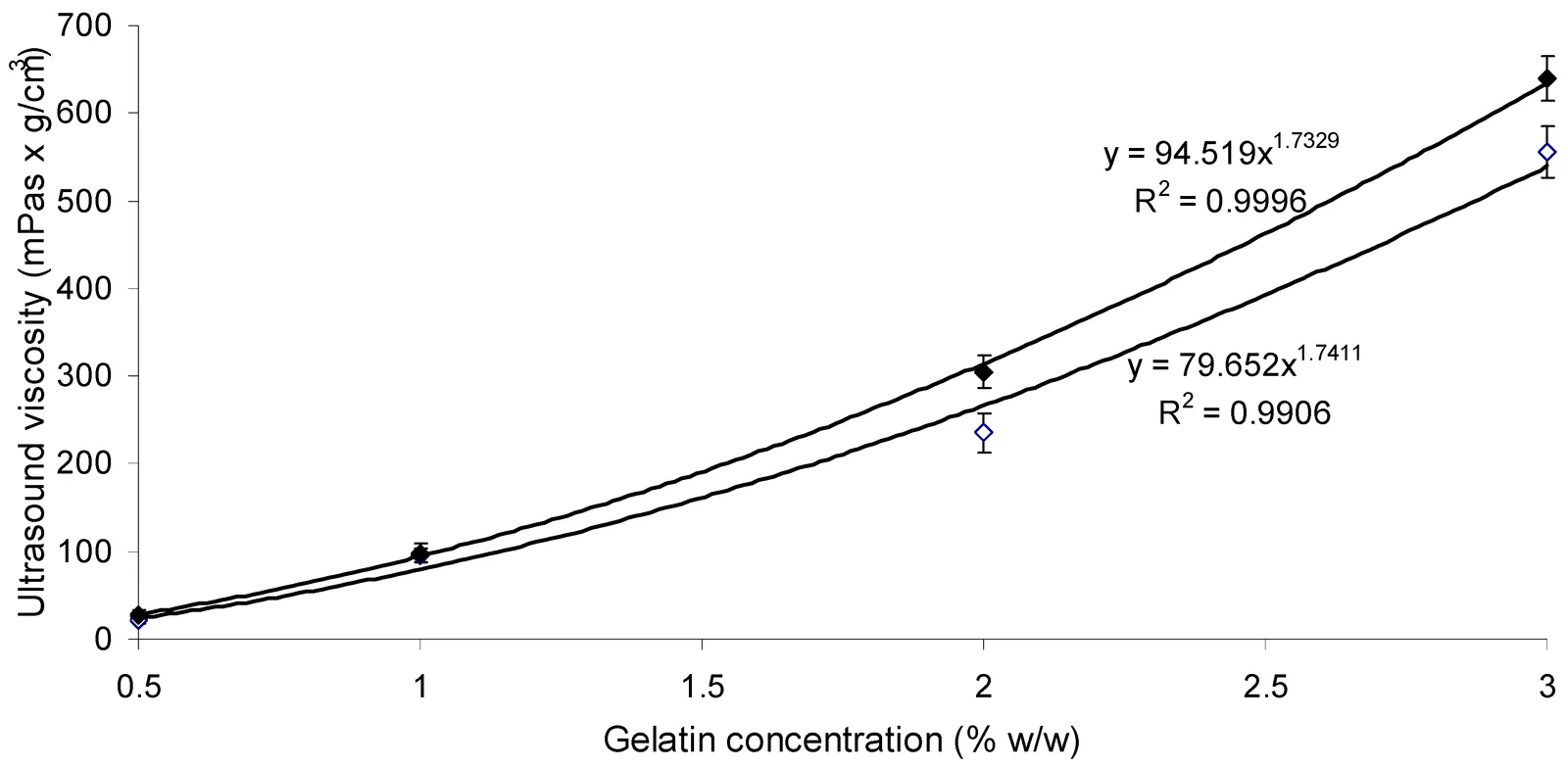
Influence of gelatin concentration in mixed gels with casein on ultrasound viscosity (open symbols—blended/aerated). Error bars represent standard deviations calculated from three independent batches of mixed gels, with six samples per batch.

Similar results were obtained using an ultrasound viscometer (Figure 3).

This instrument makes it possible to measure the viscosity of solid materials. Gelatin increased the viscosity of the gels according to a power-law relationship. Lower viscosity values were observed for the blended/aerated gels, but analysis of the power-law functions provides additional information. The coefficient “a” in the power law function:

ultrasound viscosity = a (gelatin concentration)^n^

**Figure 4:**
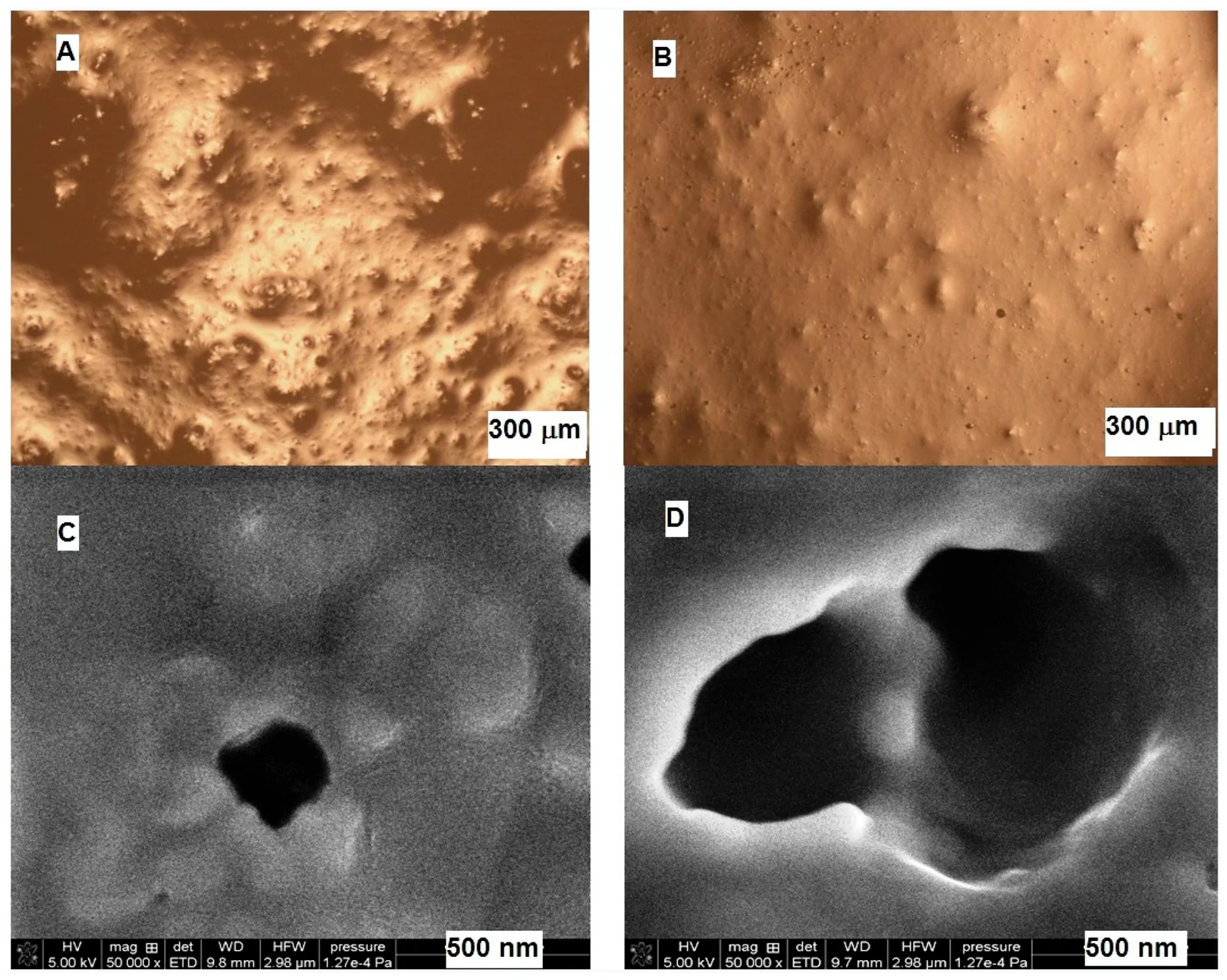
Representative micrographs of mixed gels containing 3% gelatin (top: confocal microscopy; bottom: scanning electron microscopy). Samples (**A**,**C**) were milled; samples (**B**,**D**) were subjected to blending/aeration.

Gelatin concentration was associated with increased ultrasound-viscosity values, which followed a power-law relationship. Lower values were measured for the blended/aerated gels. In the power-law equation, ultrasound viscosity = a × (gelatin concentration)^n^, the coefficient a was lower for the blended/aerated samples. This difference may partly reflect air incorporation, because air affects ultrasonic-wave propagation and the measured response [8,9]. The exponent n describes how steeply the measured value increases with gelatin concentration; its higher value for the blended/aerated gels describes a steeper concentration dependence but does not, by itself, demonstrate a stronger gel network. Differences in the micrographs (Figure 4) are observations of morphology only. The images do not establish that the visible features are gelatin aggregates or that blending/aeration caused their dissociation. The possibility that mechanical treatment altered gelatin-rich domains or their distribution remains a hypothesis requiring direct verification [10].

### 2.3. Textural Properties

**Figure 5:**
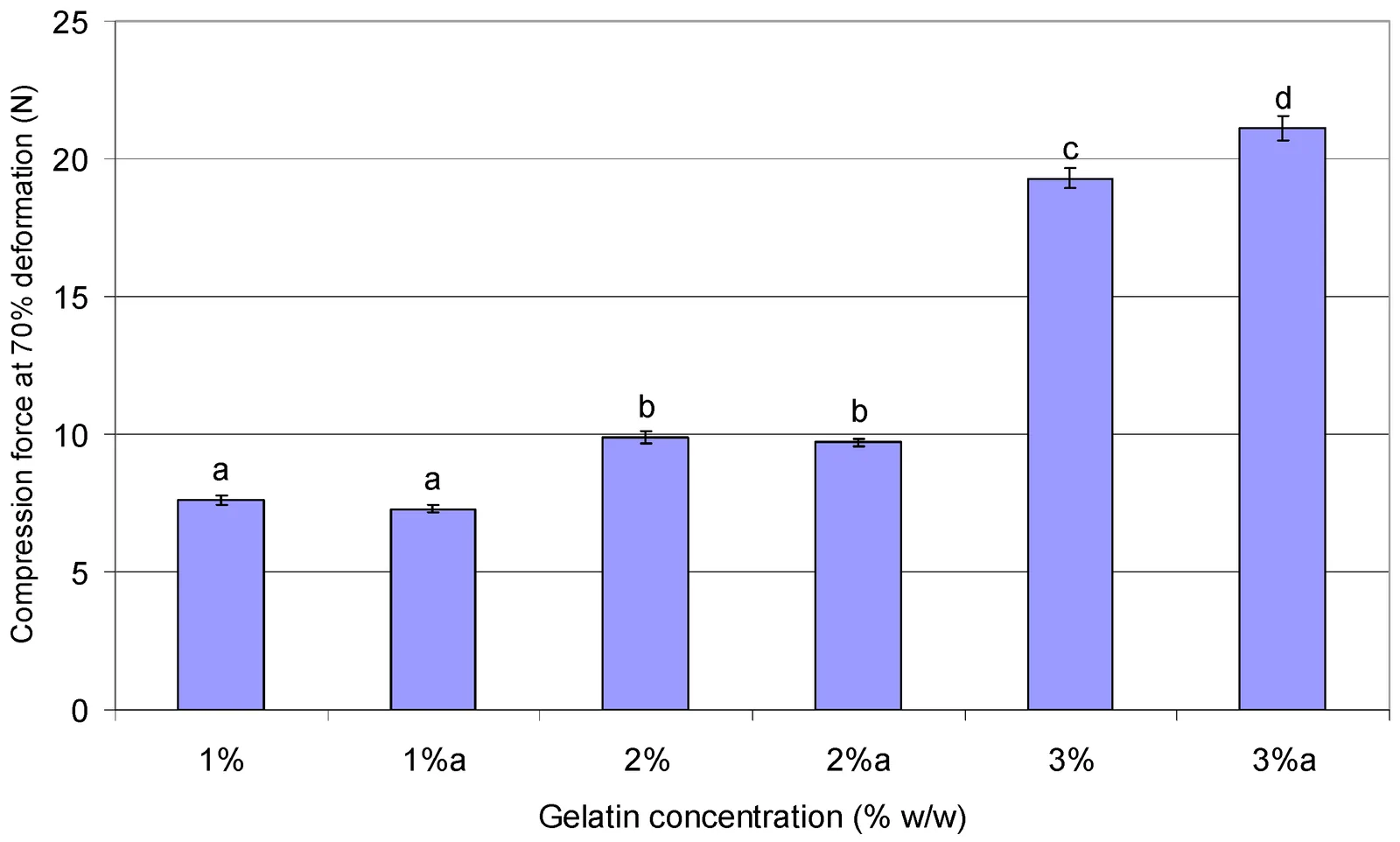
Hardness of the twaróg–gelatin gels at gelatin concentrations of 1%, 2%, and 3%, with and without blending/aeration treatment. Measurements at 0.5% gelatin were not available. Data are based on three independent batches of mixed gels, with six samples per batch. a–d Means with different letters are significantly different (*p* ≤ 0.05).

**Figure 6:**
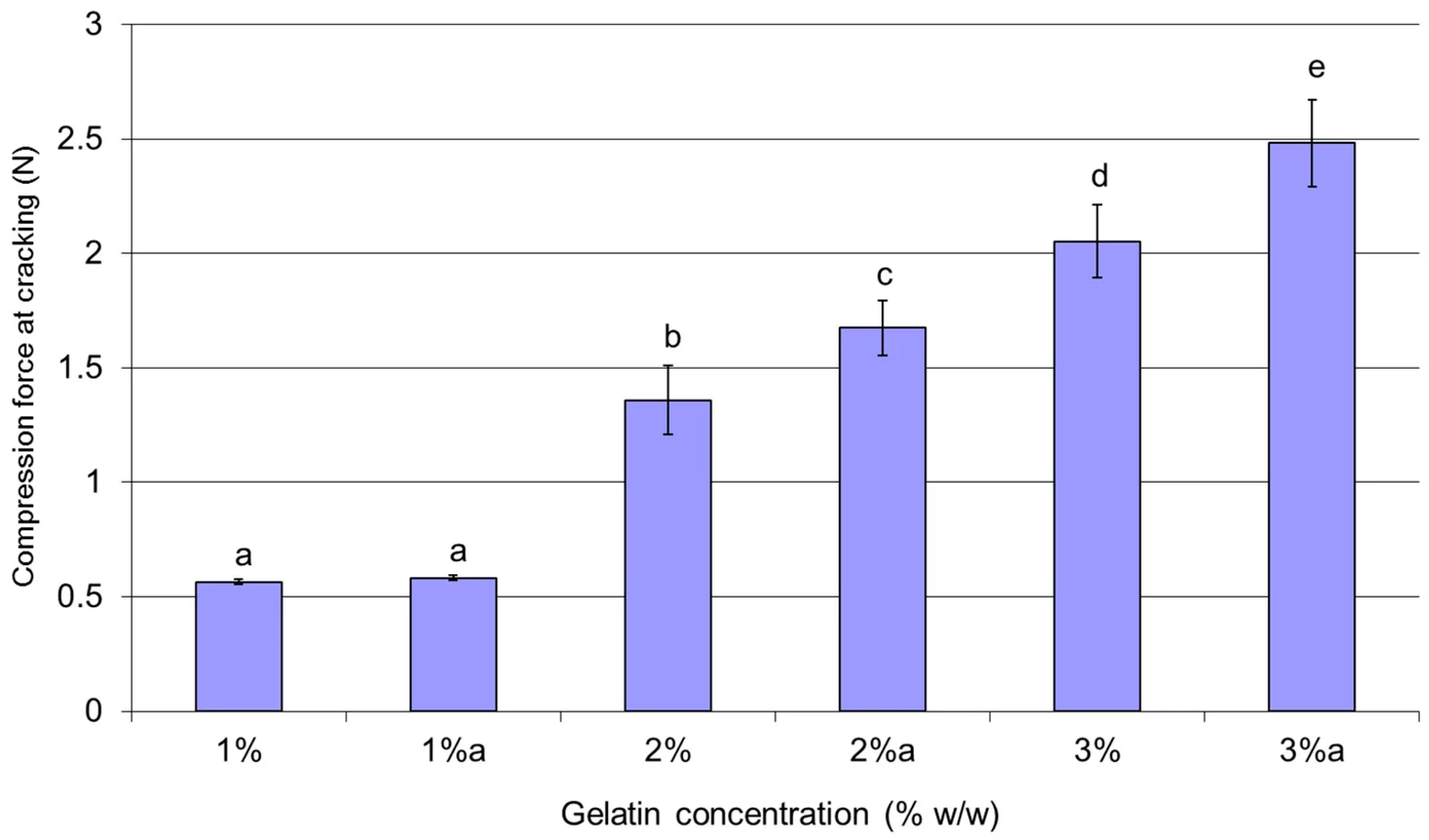
Compression force at cracking of the twaróg–gelatin gels at gelatin concentrations of 1%, 2%, and 3%, with and without blending/aeration treatment. Measurements at 0.5% gelatin were not available. Data are based on three independent batches of mixed gels, with six samples per batch. a–e Means with different letters are significantly different (*p* ≤ 0.05).

The textural properties are presented in Figure 5 and Figure 6.

The maximum compression force at 70% deformation was used as an operational measure of gel hardness. It increased with increasing gelatin concentration (Figure 5). At gelatin concentrations of 1% and 2%, blending/aeration was not associated with a detectable change in this force, whereas at 3% gelatin the treated samples had a higher value. The cracking force (the first peak in the force–distance curve; Figure 6) was higher in blended/aerated samples at all tested gelatin concentrations. These textural changes may be related to differences in the structure of the mixed gels, but the current measurements do not identify a specific molecular mechanism. In addition, because total solids were kept constant, higher gelatin concentrations coincided with lower casein fractions (Section 3.3); the trends therefore cannot be attributed to gelatin concentration alone. Tomczyńska-Mleko et al. [11] reported an initial fracture peak followed by a second peak for pure gelatin gels. The mixed gels in the present study showed smoother force–distance curves and a monotonic increase in force after the initial fracture. This response may be associated with the viscoelastic and adhesive characteristics of the mixed gels.

### 2.4. Attenuated Total Reflectance Fourier-Transform Infrared (ATR-FTIR) Spectroscopy

**Figure 7:**
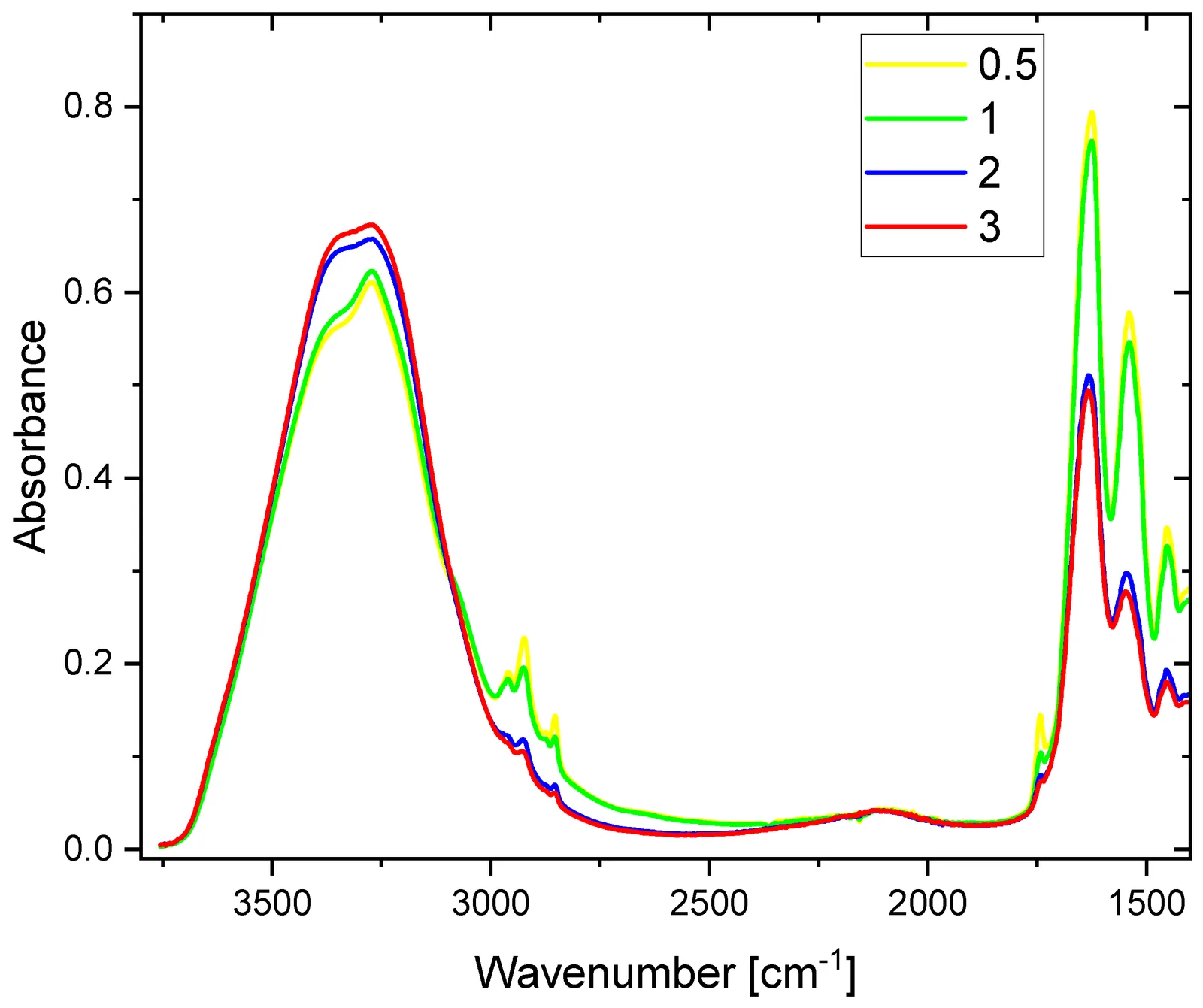
ATR-FTIR spectra of milled mixed casein–gelatin gels containing different gelatin concentrations (yellow: 0.5%; green: 1%; blue: 2%; red: 3%).

**Figure 8:**
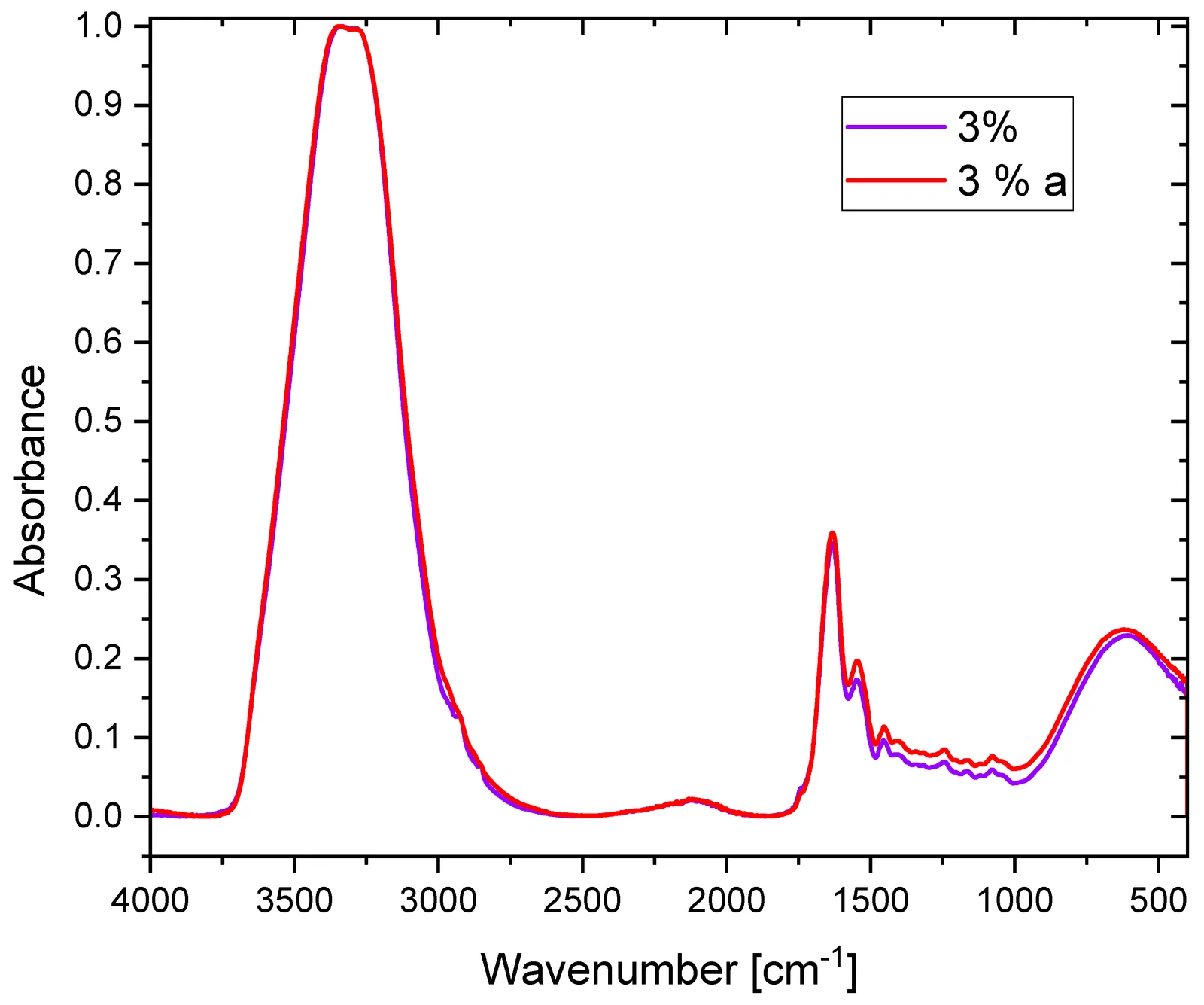
ATR-FTIR spectra of mixed casein–gelatin gels containing 3% gelatin (violet: milled sample; red: blended/aerated sample).

Figure 7 and Figure 8 show representative ATR-FTIR spectra of the mixed gels. Casein and gelatin contain similar functional groups, including peptide groups that contribute to the amide I, II, and III bands [12,13] (Figure 7).

Changes in ATR-FTIR band positions can reflect differences in the local molecular environment, including protein–water interactions, hydrogen bonding, and changes in sample composition or contact with the ATR crystal [14]. The amide II band shifted from approximately 1530 to 1545 cm^−1^ as gelatin concentration increased (Figure 7). This shift indicates a change in the environment of groups contributing to the band, but it cannot be assigned specifically to casein–gelatin interactions [15]. Amide II vibrations mainly involve N–H bending and C–N stretching and may be sensitive to hydrogen bonding, electrostatic effects, and protein conformation. Similarly, possible hydrogen bonding between casein and gelatin is one hypothesis, but the spectra do not identify the interacting components directly. Changes in hydration, gelatin–gelatin interactions, protein–water interactions, and the relative proportions of casein and gelatin may also contribute to the observed spectral differences.

The amide I band (~1600–1700 cm^−1^) originates mainly from C=O stretching vibrations in peptide groups and is sensitive to the molecular environment, including protein conformation and hydrogen bonding. In the present spectra, the band shifted from approximately 1625 to 1645 cm^−1^ as gelatin concentration increased. This observation is consistent with a change in the environment of the peptide groups, but it does not demonstrate specific casein–gelatin interactions or establish that casein secondary structure was modified [16].

A band around 3350 cm^−1^ was observed in the spectra of mixed gels containing 2% and 3% gelatin. The broad band in the ~3200–3500 cm^−1^ region is generally associated with N–H and O–H stretching vibrations and can be affected by hydrogen bonding and water content [17,18]. Its appearance or intensity may therefore reflect changes in the hydrogen-bonding environment, hydration, or the relative contributions of casein and gelatin. However, the band alone cannot establish that new hydrogen bonds formed specifically between the two proteins. Gelatin–gelatin and protein–water interactions may also contribute.

**Figure 9:**
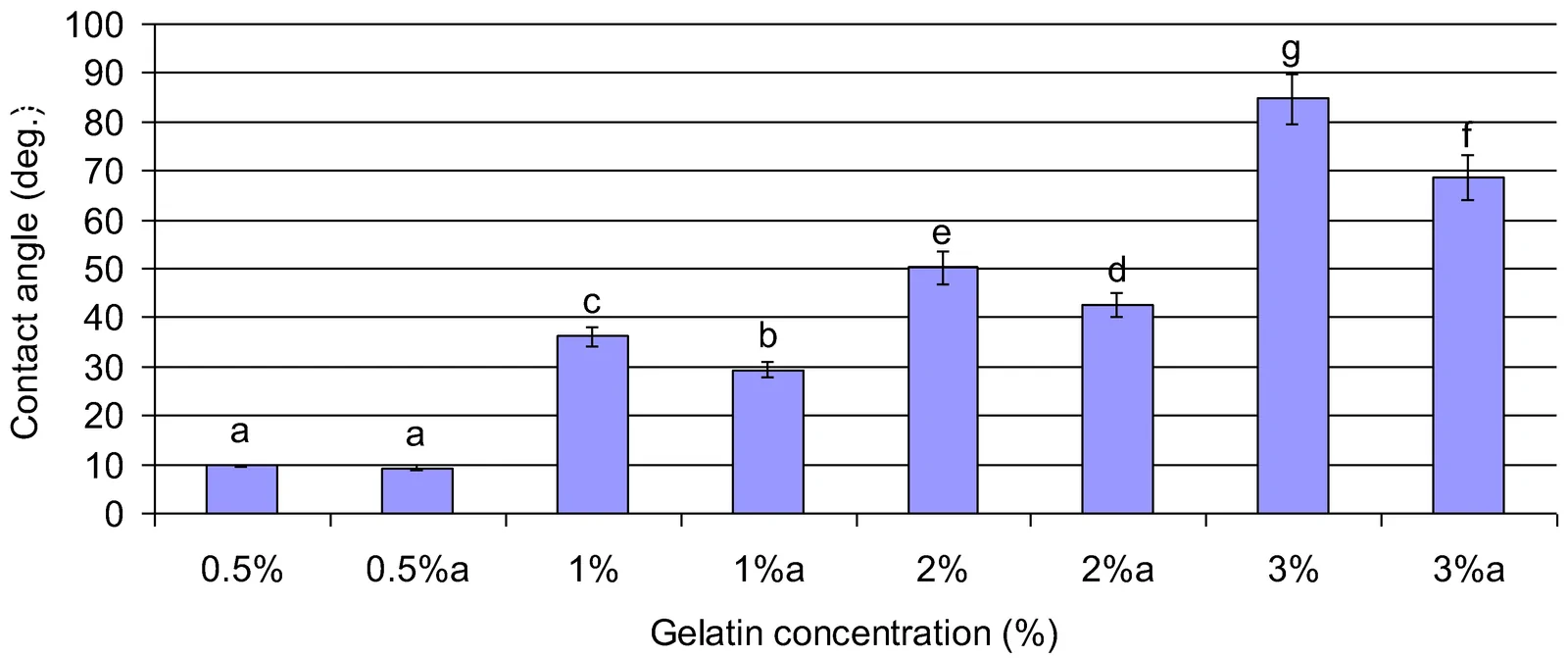
Contact angles for the surface of mixed gels obtained at different gelatin concentrations for milled samples and blended/aerated samples (a). a–g Means (three independent batches of mixed gels, with six samples per batch) with different letters are significantly different (*p* ≤ 0.05).

In the spectrum of the milled gel containing 3% gelatin, bands were visible at approximately 2923 and 2849 cm^−1^ but were less apparent in the blended/aerated sample (Figure 8). These bands are commonly assigned to C–H stretching vibrations in aliphatic groups. Their reduced intensity may reflect differences in sample composition at the surface, molecular environment, hydration, lipid distribution, or contact between the sample and the ATR crystal [19]. Because blending and aeration were performed simultaneously, the contribution of each process cannot be distinguished [20]. Although changes in the distribution of gelatin-rich domains or in the exposure of hydrophobic groups are possible explanations, the spectra do not establish these mechanisms [21,22]. Likewise, the micrographs do not provide direct evidence that blending/aeration dissociated gelatin aggregates or increased casein–gelatin interactions. Air incorporation can also alter sample contact with the ATR crystal and affect the measured signal [23]. The lower water contact angle observed after blending/aeration (Figure 9) indicates a change in surface wetting behavior, but it does not identify the molecular cause of that change [24,25].

### 2.5. Gel Surface Topography

**Figure 10:**
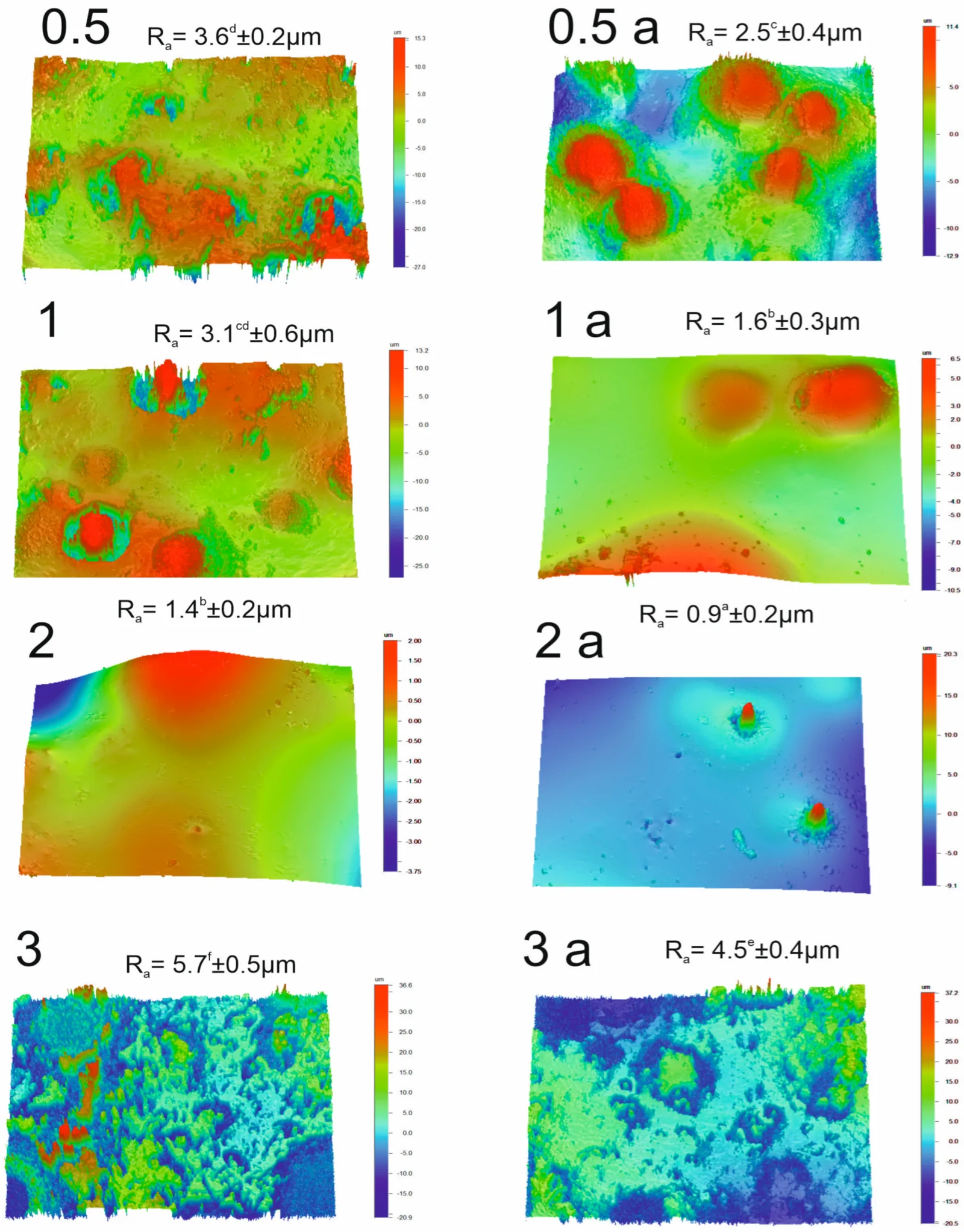
Surface roughness of mixed gels at different gelatin concentrations, with and without blending/aeration. The letter “a” denotes blended/aerated samples. The mapped surface area was 1.3 × 0.9 mm; the color scale indicates peak-to-valley height differences. a–f Means (three independent batches of mixed gels, with six samples per batch) with different letters are significantly different (*p* ≤ 0.05).

Figure 10 shows that the blending/aeration treatment was associated with lower surface roughness at gelatin concentrations of 0.5–2.0%, whereas roughness increased at 3% gelatin. The Ra value (arithmetic average roughness) represents the average absolute deviation of surface irregularities from a mean line. Shahbazi et al. [26] reported lower surface roughness in a modified starch hydrogel than in the untreated gel. In the present study, the observed differences describe surface topography; their molecular or structural origin cannot be determined from profilometry alone. Whether lower roughness improves the sensory properties of a curd-bar product remains to be tested.

### 2.6. Static Multiple Light Scattering

**Figure 11:**
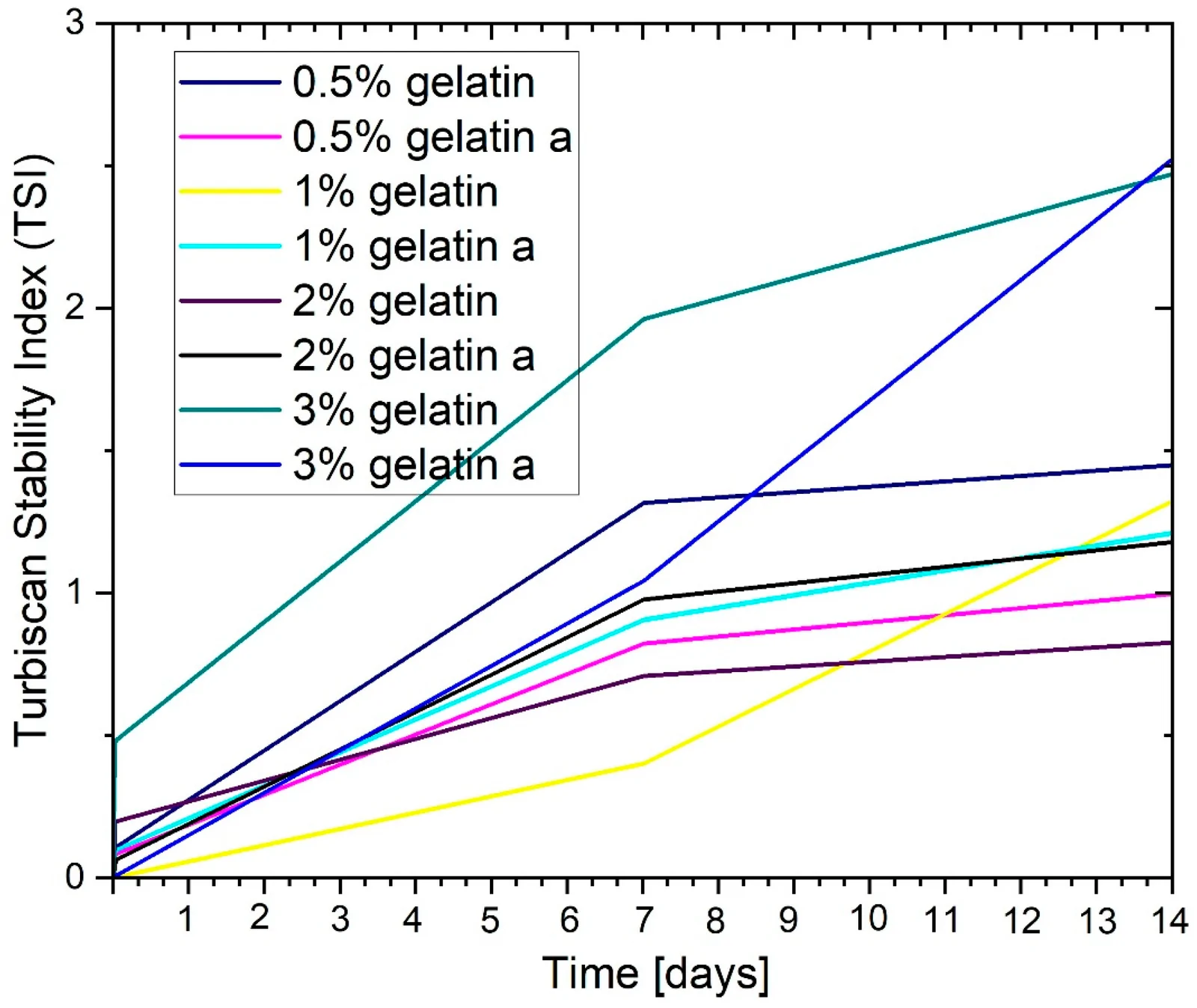
Changes in the Turbiscan Stability Index (TSI) of mixed casein–gelatin gels during storage at 7 °C. The letter “a” denotes blended/aerated samples. Each profile represents one independently prepared sample; comparisons are descriptive.

Figure 11 shows the changes in the Turbiscan Stability Index (TSI) of the mixed casein–gelatin gels during refrigerated storage at 7 °C [27]. The TSI increased over time for every formulation, indicating cumulative changes in the transmission and backscattering profiles. After 14 days, the measured TSI values were below approximately 2.6. Turbiscan measurements were obtained from one independently prepared sample per formulation, so differences between profiles and formulations are descriptive only and were not tested statistically. The values should not be interpreted as statistically demonstrated differences in physical stability or as evidence of product shelf life.

Among the measured profiles, the milled gel containing 2% gelatin had the lowest final TSI (approximately 0.82), increasing from approximately 0.70 after 7 days to 0.82 after 14 days (Figure 11). The blended/aerated gel containing 0.5% gelatin had a final TSI of approximately 0.99. At 1% gelatin, the final TSI values were approximately 1.31 for the milled sample and 1.20 for the blended/aerated sample. At 2% gelatin, the final TSI was approximately 1.17 for the blended/aerated sample. These numerical differences describe the individual profiles and should not be taken as statistically established differences between formulations. The direction and magnitude of the apparent association with blending/aeration varied with gelatin concentration.

The largest final TSI values were observed in the profiles for gels containing 3% gelatin: approximately 2.46 for the milled sample and 2.52 for the blended/aerated sample. The milled sample showed a relatively rapid increase during the first 7 days, whereas the blended/aerated sample showed a larger increase during the second week. Transmission through the bulk remained very low, consistent with the opacity of the concentrated protein gels. Changes in the profiles were mainly associated with variations in backscattering rather than a clearly defined clarification or sedimentation front. Such changes may be consistent with rearrangement of scattering structures or redistribution of the dispersed and continuous phases; however, TSI alone cannot distinguish network contraction, aggregation, and syneresis unequivocally [27]. Because only one sample per formulation was monitored, these observations do not support statistical comparisons or conclusions about improved shelf life. They show that mechanical strength and the measured optical changes during storage are different properties and should be evaluated separately.

### 2.7. Correlation Between Gelatin Concentration, Blending/Aeration and Physicochemical Properties of Mixed Gels

Table 1 presents Pearson’s correlation coefficients between gelatin concentration, blending/aeration, and the physicochemical properties of the mixed gels. It also shows correlations among all measured properties.

Blending/aeration was positively associated with cracking force and negatively associated with complex modulus, surface roughness, and contact angle, although these correlation coefficients were generally low. Gelatin concentration was strongly associated with several measured properties. Higher gelatin concentrations coincided with increased rheological parameters and greater hardness. However, total solids were held constant, so increasing gelatin concentration also decreased the casein fraction (Section 3.3). These associations should therefore be interpreted as effects of formulation composition rather than as isolated effects of gelatin alone. The contact angle increased with gelatin concentration, potentially reflecting changes in surface composition and organization; the present measurements do not establish a specific molecular explanation. Surface roughness decreased at gelatin concentrations of 0.5–2.0% in the blending/aeration-treated samples (Table 1).

### 2.8. Two-Way Analysis of Variance (ANOVA)

Table 2 presents the two-way analysis of variance (ANOVA), which was used to assess the main effects of blending/aeration treatment and gelatin concentration and their interaction. Statistical significance was set at *p* < 0.05. A significant interaction indicates that the association between gelatin concentration and a measured response differs depending on whether the sample underwent blending/aeration. Because blending and aeration occurred simultaneously, this factor represents the combined treatment and does not separate the effects of shear from those of air incorporation.

Gelatin concentration had a statistically significant effect on all variables reported in Table 2. The blending/aeration treatment had statistically significant effects on all variables except hardness. The interaction between gelatin concentration and blending/aeration was statistically significant for all variables except surface roughness.

## 3. Materials and Methods

### 3.1. Materials

Milk containing 3.2% (*w*/*w*) fat was used to produce fresh white cheese (twaróg) (Mlekpol, Grajewo, Poland). It was centrifuged to reduce the fat content to 0.1% (*w*/*w*). Type A gelatin from pig skin (Dr. Oetker Polska, Gdańsk, Poland) was used; the pH of a 3% solution was 5.52. The isoelectric point of gelatin solution was 9.2. It was determined using a Zetasizer Nano ZS (Malvern Panalytical, Malvern, UK) [6].

### 3.2. Fresh White Cheese Production

Twaróg was produced on a semi-technical scale from milk containing 0.1% (*w*/*w*) fat. The milk was pasteurized at 90 °C for 2 min. After cooling to 26 °C, a mesophilic DVS LD starter culture was added (Chr. Hansen, Hoersholm, Denmark). The starter culture contained the following bacteria: *Lactococcus lactis* subsp. Lactis, *Lactococcus lactis* subsp. cremoris, *Leuconostoc mesenteroides* subsp. cremoris, *Lactococcus lactis* subsp. lactis biovar diacetylactis. Twaróg curd was obtained by coagulating milk at 20–25 °C for 14 h. The curd was cut into 2 × 2 × 2 cm cubes and heated at 35 °C for 35 min. After cooling to room temperature, the whey was separated, and the cheese was pressed [28]. The protein, fat, and dry matter contents of the obtained twaróg were 19.6%, 0.47%, and 23.4%, respectively [29,30].

### 3.3. Preparation of Mixed Casein–Gelatin Gels

**Figure 12:**
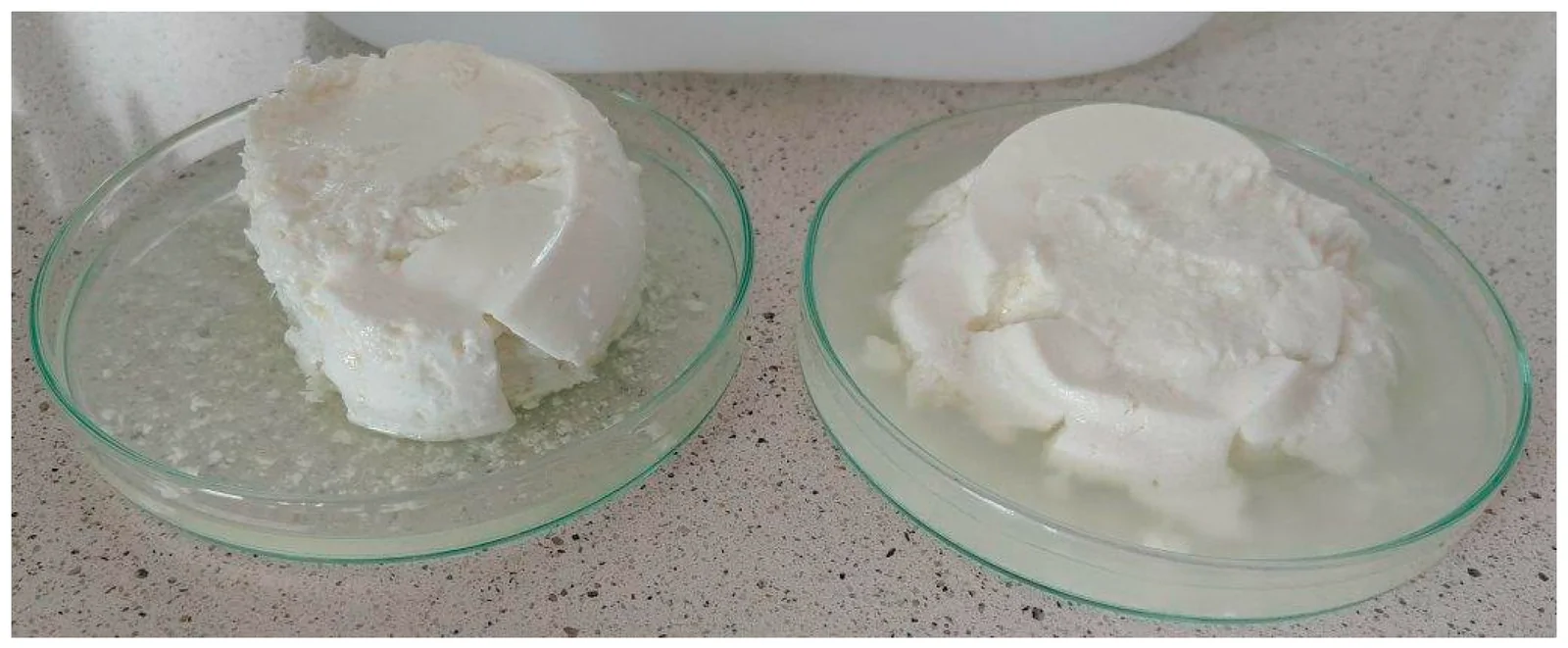
Sample without gelatin addition (the (**left**) image shows the blended/aerated sample).

Gelatin was dissolved in distilled water at 60 °C using a magnetic stirrer with an electric heater. The gelatin solution was cooled to approximately 40 °C and added to twaróg to obtain final gelatin concentrations of 0.5%, 1.0%, 2.0%, or 3.0% (*w*/*w*). The total solids content was kept constant at 21.5%. Based on the measured dry-matter content of twaróg (23.4%), the formulations per 100 g of final mixture were calculated as follows: 89.74 g twaróg, 0.50 g gelatin, and 9.76 g added water for the 0.5% gelatin formulation; 87.61 g twaróg, 1.00 g gelatin, and 11.39 g added water for the 1.0% formulation; 83.33 g twaróg, 2.00 g gelatin, and 14.67 g added water for the 2.0% formulation; and 79.06 g twaróg, 3.00 g gelatin, and 17.94 g added water for the 3.0% formulation. These amounts are calculated from the reported twaróg dry matter and assume that the gelatin powder contributes to the total solids. Because total solids were held constant, increasing gelatin concentration simultaneously decreased the casein fraction (Table 3); consequently, changes in the measured properties may reflect changes in the relative proportions of casein and gelatin. The mixture was ground for 10 min in a mortar. In a second set of experiments, the samples were additionally mixed for 2 min at 800 rpm using a Zelmer ZHB4555S hand blender (Zelmer, Warsaw, Poland). This treatment simultaneously reduced particle/cluster size and incorporated air. The effects of mechanical blending and aeration therefore cannot be separated in the present design. Mixed gels were stored in beakers at 7 °C for 24 h. Three independent batches were prepared for each formulation. Most analyses could not be performed on the sample without gelatin because it did not form a self-supporting gel and showed substantial syneresis after 24 h at 7 °C (Figure 12). The absence of a 0% gelatin control limits the establishment of a baseline for comparisons with the gelatin-containing formulations.

Table 3 presents the calculated composition and pH of the mixed gels. The pH values were similar for samples with and without blending/aeration. A small increase in pH was observed with increasing gelatin concentration.

### 3.4. Characterization of the Gels

#### 3.4.1. Texture Analysis—Compression

Cylindrical samples (10 mm high, 10 mm diameter) were cut and equilibrated to 20 °C. The cylinders were compressed to 70% deformation using a TAXT2i texturometer (Stable Micro Systems, Haselemere, UK) equipped with a P/75 compression platen (75 mm diameter) at a speed of 1.0 mm/s. Three independent batches of mixed gels were prepared and six samples per batch were analyzed. The results were averaged.

#### 3.4.2. Ultrasound Viscosity Measurements

The probe was immersed in the sample and the viscosity × density values (mPas·g/cm^3^) were measured. All the measurements were made using an ultrasound viscometer Unipan type 505 (UNIPAN, Warsaw, Poland). Three independent batches of mixed gels were prepared and six samples per batch were analyzed. The results were averaged.

#### 3.4.3. Dynamic Oscillatory Measurements

Viscoelastic properties (G*—complex modulus, tan δ) of the obtained mixed gels were measured using a Kinexus Lab+ dynamic rheometer (Malvern Instruments Limited, Malvern, UK). A serrated parallel-plate geometry (35 mm diameter, 2 mm gap size) was applied to minimize sample slippage. The gel was cut to a 2 mm high sample using a micrometer screw and then transferred into the lower plate of the rheometer. Oscillatory frequency sweeps in the range 0.1–10 Hz at 21 °C were recorded. All the measurements were taken at 1% strain, which was in the linear viscoelastic range determined previously by the strain sweep. Three independent batches of mixed gels were prepared and six samples per batch were analyzed. The results were averaged.

#### 3.4.4. Infrared Attenuated Total Reflectance (ATR-FTIR) Spectroscopy

ATR-FTIR spectra were obtained using a Nicolet iS10 FTIR spectrometer (ThermoFisher Scientific, Waltham, MA, USA). At the beginning of the research, a background spectrum was recorded. The samples were placed as plate-shaped samples on the ATR crystal, because changes in surface chemical groups were of primary interest. The absorbance spectrum (4000–400 cm^−1^) was recorded for each sample. Each spectrum was based on 32 scans. Three independent batches of mixed gels were prepared, and spectra were recorded for each independent replicate. Obtained spectra were very similar and representative spectra for mixed gels were presented. Spectra processing and comparison were performed using the OMNIC-9 software (ThermoFisher Scientific, Waltham, MA, USA).

#### 3.4.5. Confocal Microscopy

The samples were analyzed using an Axiovert 200 M confocal microscope equipped with the LSM 5 Pascal laser scanning module (Carl Zeiss, Göttingen, Germany). They were deposited on a microscope slide and a cover slide was used to minimize air pockets. The samples were visualized using a diode laser with a wavelength of 405 nm and 5× and 50× objectives.

#### 3.4.6. Scanning Electron Microscopy (SEM)

The samples were dried at 45 °C in a laboratory drier for 24 h, and fixed in a 2.5% (*w*/*w*) solution of glutaraldehyde in a 0.1 M sodium cacodylate buffer. Then the samples were dehydrated through serial dilutions of ethanol and acetone, and dried at the critical point in liquid carbon dioxide. The samples were coated with pure gold in a vacuum evaporator EMITECH K550x (Emitech, Ashford, UK) and were observed using a scanning electron microscope JEOL JXA-8230 (Tokyo, Japan).

#### 3.4.7. Contact Angle Measurements

Advancing and receding contact angles of ultrapure water (Milli-Q (18.2 mΩ)) on the sample surfaces were measured using a contact angle meter (GBX, Tallaght, Ireland). The contact angle meter was equipped with a digital camera and temperature/humidity-controlled measuring chamber. All measurements were conducted at 20 °C and 50% relative humidity. A 6 µL droplet from a syringe was deposited on the sample surface by means of an automatic deposition system. The advancing contact angle was evaluated from the droplet shape by the Win Drop++ computer program (GBX, Tallaght, Ireland). A volume of 2 µL was withdrawn from the droplet into the syringe and the receding contact angle was determined using the software. The advancing and receding contact angles were measured for 10 droplets for each sample. Three independent batches of mixed gels were prepared and six samples per batch were analyzed. The results were averaged.

#### 3.4.8. Gel Surface Topography

Gel surfaces were investigated using an optical profilometer GT Contour Surface Metrology (Veeco, Tucson, AZ, USA). The surface topography was characterized in the range from sub-nanometer to 10 mm. The surface roughness was calculated using the Vision64 computer program (Veeco, Tucson, AZ, USA). Three independent batches of mixed gels were prepared and six samples per batch were analyzed. The results were averaged.

#### 3.4.9. Static Multiple Light Scattering

The physical stability of mixed casein–gelatin gels during refrigerated storage was monitored by static multiple light scattering using a Turbiscan Lab analyzer (Formulaction, Toulouse, France). Undiluted samples were placed in cylindrical glass measurement cells and analyzed at 7 °C. The instrument scanned the sample height using near-infrared light and recorded transmission (T) and backscattering (BS) profiles as functions of height and storage time. This method monitors optical changes that may be associated with migration of particles or protein aggregates, changes in the size of scattering structures, and redistribution of dispersed and continuous phases [31]. The TSI was calculated by the instrument software from cumulative changes in transmission and backscattering profiles. Lower TSI values indicate smaller changes in the optical profiles during the measurement period; they do not, on their own, establish product shelf life or identify a specific physical mechanism. TSI values after 14 days were compared descriptively. One independently prepared sample per formulation was monitored continuously for 14 days. Sequential scans of the same vial were not treated as independent replicates, and the TSI profiles were not subjected to inferential statistical analysis.

### 3.5. Statistical Analysis

Pearson correlation coefficients were calculated, and two-way analysis of variance (ANOVA) was performed. Blending/aeration was coded as a binary variable (0 = no treatment, 1 = blending/aeration). Three independently prepared batches were analyzed across all experimental variants. Six analytical replicate measurements were performed for each batch and experimental variant. The six measurements were averaged to obtain one mean value per batch and variant. These batch-level means were used for statistical analysis, with the batch treated as the experimental unit.

Pearson correlation coefficients were calculated using mean values from three independently prepared batches. The number of observations (*n*) varied depending on the pair of properties analyzed and the availability of measurements across gelatin concentrations. Correlations between properties measured at all four gelatin concentrations (0.5%, 1%, 2%, and 3%) were based on 24 observations, whereas correlations involving hardness or cracking force were based on 18 observations, corresponding to the three concentrations (1%, 2%, and 3%) for which these measurements were available. Two-way ANOVA was also performed for hardness and cracking force, using only the three gelatin concentrations (1%, 2%, and 3%) for which measurements were available. The corresponding F- and *p*-values were calculated using this three-concentration experimental design. For the remaining properties, all four gelatin concentrations (0.5%, 1%, 2%, and 3%) were included.

Tukey’s post hoc test was used to identify statistically significant differences between means at *p* < 0.05. All statistical analyses were performed using Statistica 13.3 (StatSoft, Warsaw, Poland).

## 4. Conclusions

The results show that gelatin concentration and the combined blending/aeration treatment were associated with changes in the rheological, textural, microstructural, and surface properties of mixed twaróg–gelatin gels. Increasing gelatin concentration was associated with stronger viscoelastic and textural responses. Because total solids were maintained at 21.5%, higher gelatin concentrations also corresponded to lower casein fractions; the observed trends therefore reflect changes in the relative proportions of both protein components and cannot be attributed to gelatin concentration alone. ATR-FTIR band shifts indicate changes in the local molecular environment but do not demonstrate specific casein–gelatin interactions or changes in casein secondary structure. The blending/aeration treatment was associated with changes in microstructure, lower surface roughness, and a lower water contact angle. The observed effects may reflect several factors, but the present measurements do not isolate their individual contributions. Turbiscan profiles showed formulation-dependent optical changes during refrigerated storage. The milled gel containing 2% gelatin had the lowest final TSI among the measured profiles; however, as only one independently prepared sample per formulation was monitored, these results are descriptive and do not establish statistically significant differences in stability or improved shelf life. Mechanical strength and storage-related optical changes should therefore be treated as distinct properties. The mixed gels may have potential for refrigerated curd-cheese snack products, but syneresis, shelf life, sensory acceptability, and behavior above refrigeration temperature must be evaluated before practical application can be established. Future work should separate mechanical mixing from aeration, use replicated storage-stability measurements, and apply complementary methods to investigate possible molecular interactions. Further studies could also compare other hydrocolloids and gelatin combinations with micellar casein or sodium caseinate.

## Figures and Tables

**Table 1:** Pearson’s correlation coefficients between measured physicochemical properties of mixed gels at different gelatin concentrations with or without blending/aeration treatment. Correlations between properties measured at all four gelatin concentrations (0.5%, 1%, 2%, and 3%) were based on 24 observations, whereas correlations involving hardness or cracking force were based on 18 observations, corresponding to the three concentrations (1%, 2%, and 3%) for which these measurements were available.

	Blending/Aeration (Yes or No)	Gelatin Conc. (%)	Surface Roughness—R_a_ (μm)	Contact Angle	Hardness (N)	Cracking (N)	Ultrasound Viscosity (mPas × gcm^−3^)	Complex Modulus at 1 Hz (Pa)
Surface roughness—R_a_ (μm)	−0.34	−0.42 *						
Contact angle	−0.16	0.96 **	0.49 *					
Hardness (N)	0.07	0.91 **	0.79 **	0.90 **				
Cracking (N)	0.17	0.96 **	0.50 *	0.82 **	0.87 **			
Ultrasound viscosity (mPas × gcm^−3^)	−0.09	0.97 **	0.57 **	0.96 **	0.93 **	0.90 **		
Complex modulus at 1 Hz (Pa)	−0.32	0.88 **	0.46 *	0.92 **	0.65 **	0.68 **	0.91 **	
tan δ	−0.05	−0.95 **	−0.17	−0.90 **	−0.78 **	−0.96 **	−0.86 **	−0.79 **

* *p* < 0.05; ** *p* < 0.01.

**Table 2:** Results of two-way analysis of variance (ANOVA) for the effects of gelatin concentration and blending/aeration treatment on the measured properties. ANOVA was performed using the mean of six analytical replicates for each independently prepared batch and formulation. The experimental unit was the independently prepared batch (*n* = 3 per formulation). For hardness and cracking force, the analysis included only the three gelatin concentrations (1%, 2%, and 3%) for which measurements were available; for all other properties, the analysis included four gelatin concentrations (0.5%, 1%, 2%, and 3%). For hardness and cracking force, the degrees of freedom were 1 for blending/aeration, 2 for gelatin concentration, 2 for their interaction, and 12 for residual error (total df = 17). Statistically significant effects are shown in bold.

	Blending/Aeration (Yes or No)			Gelatin Conc. (%)			Blending/Aeration (Yes or No) × Gelatin Conc. (%)		
	Test F	F	*p*	Test F	F	*p*	Test F	F	*p*
Surface roughness—R_a_ (μm)	4.49	48.7	**3.13 × 10^−6^**	3.24	115	**4.72 × 10^−11^**	3.24	1.85	0.18
Contact angle	4.49	40.8	**8.87 × 10^−6^**	3.24	539	**2.81 × 10^−16^**	3.24	7.12	**3.00 × 10^−3^**
Hardness (N)	4.75	2.32	0.15	3.89	481	**3.50 × 10^−12^**	3.89	4.12	**4.34 × 10^−2^**
Cracking (N)	4.75	15.1	**2.16 × 10^−3^**	3.89	432	**6.61 × 10^−12^**	3.89	4.90	**2.78 × 10^−2^**
Ultrasound viscosity (mPas × gcm^−3^)	4.49	31.3	**4.06 × 10^−5^**	3.24	1210	**4.57 × 10^−19^**	3.24	8.47	**1.00 × 10^−3^**
Complex modulus at 1 Hz (Pa)	4.49	11.4	**3.00 × 10^−24^**	3.24	28.7	**4.74 × 10^−30^**	3.24	4137	**2.52 × 10^−23^**
Loss tangent	4.49	36.8	**1.64 × 10^−5^**	3.24	4213	**2.18 × 10^−23^**	3.24	52.8	**1.61 × 10^−8^**

**Table 3:** Calculated composition of mixed gels and pH measured after storage at 7 °C for 24 h. pH values are presented as mean ± standard deviation (SD) calculated from three independent batch means (*n* = 3). Each batch mean was obtained from six analytical samples.

Gelatin Concentration (% *w*/*w*)	pH		Total Solids (%)	Casein (%)	Fat (%)
	No Blending/Aeration	Blending/Aeration			
0.5	4.50 ± 0.12	4.51 ± 0.23	21.5	17.6	0.42
1.0	4.55 ± 0.34	4.57 ± 0.31	21.5	17.2	0.41
2.0	4.58 ± 0.13	4.59 ± 0.19	21.5	16.3	0.39
3.0	4.66 ± 0.28	4.60 ± 0.31	21.5	15.4	0.37

## Data Availability

The raw data supporting the conclusions of this article will be made available by the authors on request.
